# The Pathway between Social Dominance Orientation and Drop out from Hierarchy-Attenuating Contexts: The Role of Moral Foundations and Person-Environment Misfit

**DOI:** 10.3390/bs13090712

**Published:** 2023-08-28

**Authors:** Alessio Tesi, Daniela Di Santo, Antonio Aiello

**Affiliations:** Department of Political Sciences, University of Pisa, Via F. Serafini, 3, 56126 Pisa, Italy; daniela.disanto@unipi.it (D.D.S.); antonio.aiello@unipi.it (A.A.)

**Keywords:** social dominance, moral foundations, person-environment fit, intergroup relations, social hierarchies

## Abstract

The present study examines the role of individuals’ preference for unequal intergroup relations in exacerbating a process of differential attrition from organizations that value intergroup equality (i.e., hierarchy-attenuating contexts). We proposed that people functioning within a well-recognized hierarchy-attenuating context (i.e., students of social work) who were higher on social dominance orientation (SDO) would be more likely to leave their institution through two pathways; first, people higher on SDO would have fewer moral concerns of social fairness and human harm-avoidance (i.e., individualizing); in turn, a lack of individualizing morality would stimulate a perceived person-environment misfit, ultimately increasing their intention to leave. We conducted a single cross-sectional design study involving a convenience sample of 245 undergraduate social work students. Overall, the results of the serial mediation model suggest that people higher on SDO intend to leave their organization that supports inclusive equality via reduced individualizing morality and high perceived P-E misfit. These findings contribute to understanding the role of socio-political orientations and moral beliefs in hindering proper adaptation to contexts that value egalitarian social norms, with relevant implications for individuals and groups.

## 1. Introduction

People’s behavior is influenced by both their personality and the social norms that rules what is considered desirable in a given context. People assess the congruence between who they think they are (i.e., the Self) and what they perceive to be normative in the setting [1]. This assessment may include an evaluation of the congruence or incongruence, to varying degrees, between one’s own sociopolitical attitudes and moral beliefs and the social and moral norms most commonly held and shared by other people in a context. When people’s attitudes are congruent with the social norms most held and shared in a context, people perceive a person-environment (P-E) fit [2,3], whereas when there is a mismatch between these two components, people deal with a P-E misfit [4]. The P-E misfit theory [5] encompasses several assessments of incongruity between individuals and organizations, such as values incongruence, incongruence between specific job requirements and personal abilities to meet them, and incompatibility between employees and leaders personalities. In order to study P-E incongruence, it is necessary to delineate the “field of incongruence” to make it meaningfully interpretable [5]. Based on this notion, we focused on a specific form of P-E misfit that results from the incongruence between personal and organizational socio-political orientations [6]. Specifically, drawing on social dominance theory (SDT) [7], we studied the possible consequence (i.e., behavioral intention to leave the context) that might result from a particular form of attrition; i.e., when people report high levels of social dominance orientation (SDO, i.e., the extent to which people want to support group-based hierarchies and inequalities), and when they are in environments where the organizational culture strongly values SDO-inconsistent, hierarchy-reducing social norms (i.e., social welfare). Studies [8,9] have previously confirmed that people’s level of SDO is associated with an increased perceived P-E misfit in hierarchy-attenuating contexts, and that PE misfit can subsequently lead people to express a higher willingness to leave their organization [10]. In the present study, we deepened these findings by testing the hypothesis that perceived P-E misfit could be generated by a set of moral beliefs associated with higher levels of SDO. This hypothesis is based on studies suggesting that individuals with higher levels of SDO score lower on measures of empathy and self-transcendence (i.e., universalism, benevolence) [11,12] and higher on measures of competitiveness and self-enhancement [13,14]. Thus, we expected that SDO would be associated with lower moral concerns about others (i.e., harming others, be unfair), defined as “individualizing” [15,16]. Furthermore, we tested the hypothesis that perceived P-E misfit could be elicited by a lack of moral stance—which may arise from a greater level of SDO—that does not value people in socially marginalized positions. Finally, we expected that a consequence of this P-E misfit would be a desire to withdraw from the hierarchy-attenuating context, as an attempt to reduce the dissonance associated with the experienced incongruence with the environment [17].

In summary, drawing on social dominance theory (SDT) [7], moral foundations theory [16,18,19], and P-E fit theory [2,3,20], we tested a model in which SDO increases intention to leave a social work organization that strongly values people from low-status groups [9,16], and this occurs due to lower individualizing morality and higher P-E misfit perception.

### 1.1. Social Dominance Theory and Social Dominance Orientation

SDT [7] is one of the most influential theories aimed at explaining the socio-psychological dynamics involved in the production and reproduction of social inequalities in different contexts and domains. SDT has been used as a framework to address how group-based hierarchies are produced and maintained in societies [21]. Group inequalities are created by the spread of legitimizing myths that either enhance or attenuate hierarchy., Legitimizing myths are sets of beliefs, stereotypes, and ideologies that promote (e.g., racism, sexism, nationalism, classism, meritocracy) or attenuate (e.g., support for democracy and inclusiveness) asymmetrical intergroup relations. SDT posits that support for group-based hierarchies can be regulated by a psychological orientation called SDO [22]. Irrespective of their position in social hierarchies, people with higher levels of SDO show greater endorsement of hierarchy-enhancing legitimating myths and less endorsement of hierarchy-attenuating myths. People who report higher levels of the SDO endorse a set of beliefs related to asymmetrically structured group hierarchies (i.e., “an ideal society requires some groups to be on the top and others on the bottom”) [23] and anti-egalitarian policies (“it is unjust to try to make groups equal”) [23]. In addition, people who score higher on the SDO endorse a set of beliefs related to a “competitive worldview”, in which intergroup and social relations are depicted as a zero-sum competitive game in which a gain in resources for some groups result in a loss for others [13,24]. This type of social competition reflects an evolutionary vision of society in which the strongest win and the weakest lose [7,25]. According to this notion, SDO was found positively associated with self-enhancement, achievement, and coercive power and negatively associated with empathy, emotional concern for the harm of others, and social justice [11,14,26]. Individuals higher on SDO are more likely to pursue self-enhancement goals (i.e., salary, status) even in contexts where social justice and solidarity with marginalized groups are normative [10]. Since the production of social inequalities cannot neglect the role of contextual social norms, the ways through which people can express and shape their SDO levels are influenced by social norms and by the characteristics of social contexts that can support or hinder the legitimizing myths of hierarchies [6,27,28].

### 1.2. SDO and Person-Environment (Mis)fit

To study the role of social norms, scholars have applied the principles of SDT to mesosystems such as communities and organizations [26,29,30]. To the extent that social dominance is pervasive in any social structure that includes social hierarchies, organizations are ideal settings for studying how asymmetric relationships are created and maintained [31]. Haley and Sidanius [6] proposed a distinction between hierarchy-enhancing and hierarchy-attenuating contexts based on the key norms and ideologies supported and shared within a given setting. Hierarchy-enhancing contexts are those settings that promote group-based inequalities through a differential distribution of social, political, and economic power in favor of dominant groups and against subordinate groups. For example, military and profit-maximizing organizations encompass a culture of dominance that sustains hierarchy-enhancing legitimizing myths that allocate greater social value to dominant (vs. subordinate) groups [29,30]. On the contrary, hierarchy-attenuating contexts (i.e., humanitarian, nonprofit, and social work organizations) promote and share social norms that privilege the reduction of group-based dominance and inequalities and disseminate and value hierarchy-attenuating legitimizing myths (i.e., social justice, dignity, and worth of the person).

Drawing on the person-environment (P-E) fit theory [2,3,20], studies have found that people with higher levels of SDO report high P-E fit in hierarchy-enhancing contexts [29], whereas people with lower levels of SDO report high P-E fit in hierarchy-attenuating environments [8]. As a result of P-E fit, hierarchy-enhancing and hierarchy-attenuating contexts appear as homogeneous environments in which people share the same beliefs, attitudes, and values that are consistent with their respective organizational functions. Consistent with these processes that foster ideological homogeneity within contexts, several studies [27,28,30] have highlighted that people higher on SDO are more likely to be attracted to hierarchy-enhancing careers (i.e., police, law enforcement), whereas people lower in SDO are more likely to pursue hierarchy-attenuating careers (i.e., social work).

In contrast, when levels of SDO are incongruent with the hierarchy-enhancing or attenuating function of the context, people experience a condition of P-E misfit [6,9]. Tesi and colleagues [10] found that the higher social work students were on SDO, the more they perceived a greater P-E misfit over time. In additions, those students who perceived the misfit were found to be more prone to leave the context, as a possible behavioral strategy to resolve the attrition [32]. Although the authors [10] interpreted the P-E misfit as resulting from the misalignment between students’ SDO and the mission of social work, in the present study we hypothesized and tested a model in which P-E misfit, and thus intention to leave, is best captured by moral beliefs fueled by SDO, rather than by SDO itself.

### 1.3. The Missing Link between SDO and P-E Misfit: The Individualizing Moral Foundations

Moral beliefs held by individuals could mediate the path between SDO and perceived P-E misfit. In moral foundations theory (MFT) [16,18,19,33], the range of potential human moral motivations can be grouped into a restricted number of basic moral motives. In particular, MFT [18] identified five moral foundations through which people make moral judgments about “right” and “wrong”: harm/care, fairness/reciprocity, in-group/loyalty, authority/respect, and purity/sanctity. The five dimensions were often categorized into two broader clusters of “individualizing” foundations (i.e., care and fairness), and “binding” foundations (i.e., authority, in-group loyalty, and sanctity) [16]. While the binding foundations were primarily concerned with preserving order and cohesion in human groups, the individualizing foundations were primarily concerned with protecting individuals from harm or violations of their rights and freedoms [16]. According to the theory, moral foundations are somehow derived from an evolutionarily designed and culturally refined psychological system, whereby the individualizing sub-dimensions of harm/care and fairness/reciprocity are rooted in the evolution of empathy and mutual altruism [15,18]. However, subsequent research has shown that people tend to harmonize their moral concerns with their prior intergroup beliefs and attitudes [34]. Consistently, individualizing concerns are stimulated by personal orientations pertaining to the support of social equality versus inequality [9]. Because people with higher levels of SDO appear to be less concerned about what might hurt others in subordinate positions and more concerned about what is beneficial to their self-enhancement, achievement, and power status [12,14], the greater value given to individualizing concerns could be attributed to lower levels of SDO. Conversely, this could be stated as: lower values of individualizing concerns would be attributed to higher levels of SDO [35,36]. Moreover, the SDO-driven competitive worldview and the desire to promote dominance-submission forms of intergroup relations [13] appear incompatible with the moral concerns of protecting the community by morally sanctioning harm to others and making people respect other individuals’ rights. Consistently, the endorsement of individualizing foundations has consequences for the solidarity and inclusivity of individuals in groups by promoting the acceptance of minority groups [37,38] or reporting greater prosocial behavioral tendencies [39]. In this sense, individualizing morality appears to fit into hierarchy-attenuating contexts promoting social welfare, human rights, and group equality. Furthermore, having few individualizing moral concerns might stimulate a perceived incongruence with the social norms that are mostly endorsed and shared in hierarchy-attenuating contexts. In the present study, we expected that SDO-driven low individualizing moral beliefs would foster a perceived misfit with the anti-dominant social norms pursued and shared in the study’s context.

### 1.4. The Present Study

Drawing on previous research [6,28,30], the overall aim of the present study was to examine how people with a preference for social hierarchies deal with a hierarchy-attenuating context in which social norms support inclusive equality. We conducted a single cross-sectional design study involving a sample of social work students. The contexts in which social work is taught and practiced are widely and globally recognized as hierarchy-attenuating [8,9,27]; indeed, the primary goal of social work is to promote human well-being, with particular attention to the empowerment of those who are socially, politically, and economically disadvantaged (i.e., the poor, the disabled, the elderly, immigrants). Social work supports inclusive practices and the fight against social injustice (i.e., oppression, discrimination, poverty; cf. National Association of Social Workers [NASW] Code of Ethics, 2021) [40].

In such a hierarchy-attenuating context, consistent with the literature [35,36], we first hypothesized that SDO would be negatively associated with individualizing moral beliefs. Because we hypothesized that higher levels of individualizing beliefs would be negatively associated with P-E misfit in this hierarchy-attenuating setting, we expected, in contrast, that lower individualizing moral beliefs, fostered by high levels of SDO, would promote P-E misfit. We then tested whether the sociopolitical attrition resulting from higher levels of SDO in hierarchy-attenuating contexts could be explained by less endorsement of the moral principles of care and fairness. Finally, consistent with previous research [10], we hypothesized that P-E misfit would be positively associated with drop out intentions. Therefore, we expected that people would choose to leave the organization as a possible strategy to cope with the perceived inconsistency of the P-E misfit.

**Figure 1:**
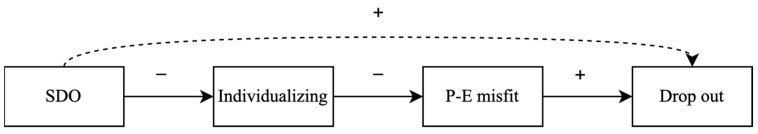
Graphical representation of the expected associations among variables. The dotted line indicates that an indirect effect of SDO on drop out is assumed.

Specifically, we examined the above hypotheses through a comprehensive model that tests the effects of SDO on drop out as serially mediated by individualizing moral beliefs and perceived P-E misfit. The conceptual model and the expected associations among variables are shown in Figure 1.

## 2. Methods

### 2.1. Participants and Procedures

To test the study’s hypotheses, we conducted a cross-sectional study. We recruited a convenience sample of social work students attending the first three years of their social work graduation program. According to many studies [9,27], social work faculties are considered hierarchy-attenuating settings. People in social work contexts are pushed to endorse and share hierarchy-attenuating legitimizing myths that promote the empowerment and well-being of people in disadvantaged positions [40].

To estimate the adequate sample size to test our serial mediation model, we used Monte Carlo power analysis tool for indirect effects [41]. Consistent with the coefficients reported in the literature [9], we assumed moderate effect sizes (all paths, r = 0.30) and set up 5000 replications and 20,000 Monte Carlo draws per replication. A minimum of 241 participants was suggested to achieve a sufficient power of 0.80.

At the beginning of the academic year, we asked 288 students to participate in the research program voluntarily. A total of 245 students (229 women, 16 men) agreed and were enrolled in the research (response rate of 85.07%). Their mean age was 22.44 years (SD = 3.62). Participants were invited to specific data collection sessions. They signed an informed consent form and completed an anonymous online self-report questionnaire.

### 2.2. Measures

#### 2.2.1. SDO

The SDO was measured by administering the Italian version [42] of the SDO_7_ scale [23]. The scale is composed of 16 item that captures the extent to which individuals support group-based dominance (i.e., “An ideal society requires some groups to be on top and others to be on the bottom”) and anti-egalitarianism (i.e., “It is unjust to try to make groups equal”). The scale presents a 7-point Likert answer format ranging from 1 = “strongly oppose” to 7 = “strongly favor”. In the present study, the scale reliability was satisfactory (Cronbach’s α = 0.84).

#### 2.2.2. Individualizing Foundations

The endorsement of individualizing moral foundations was measured by administering the Italian version [43] of the Moral Foundations Questionnaire’s (MFQ) Individualizing sub-scale [44]. The scale consisted of a 12-item measure of the extent to which people held the individualizing foundations (with subfactors of Harm/care and Fairness/reciprocity) and was composed of two parts. In the first part, participants rated the relevance (from “1 = Not at all relevant” to “6 = Extremely relevant”) of six sources of information that could be used when they make moral judgments (e.g., “Whether or not someone cared for someone weak or vulnerable”). In the second part, participants rated their agreement (from “1 = Strongly disagree” to “6 = Strongly agree”) with six moral statements (e.g., “Compassion for those who are suffering is the most crucial virtue”). In line with studies that used the broad categories of moral foundations [45], we collapsed the two subscales of harm and fairness into the higher-order variable of individualizing foundations. The scale’s internal reliability in the present study was satisfactory (Cronbach’s α = 0.74).

#### 2.2.3. P-E Misfit

The Italian adaptation of the scale proposed by Deng and colleagues [46] for the social work context [7] was used to measure P-E misfit. The scale specifically assesses incompatibility with social work values and culture. Participants answered on a 7-point Likert scale ranging from “1 = completely disagree to 7 = completely agree” to the following items, “The things that I value in life are not similar to the things that the Social Work major values”, “My personal values do not match the Social Work major culture”, “The Social Work major values and culture do not provide a good fit with the things that I value in life”. To facilitate the prompt accessibility of social norms and social representations pertaining to social work, prior to responding to the P-E misfit scale, participants read a statement (Appendix A) that had the function of priming their affiliation to a work environment characterized by a hierarchy-attenuating function. This priming procedure has been shown to be effective in improving the reliability associated with the measurement of P-E misfit [4,5]. In the present study, the scale showed satisfactory internal reliability (Cronbach’s α = 0.70).

#### 2.2.4. Drop out Intention

Students’ intention to leave the social work program was measured by adapting the turnover intention scale [47] to university contexts. The scale was composed of the following four items, “I think I made a mistake in choosing this course of study”, “I intend to leave this course of study”, “I expect to change this course of study”, “I am thinking of leaving the university to pursue something else”. The scale presented a 5-point Likert scale answer format ranging from 1 = “not true at all for me” to “totally true for me”. The scale internal reliability was satisfactory (Cronbach’s α = 0.82).

## 3. Results

### 3.1. Assessment of the Measurement Model

To evaluate the measurement model of the study, we conducted a confirmatory factor analysis (CFA) including four correlated latent variables corresponding to the item scales (SDO, individualizing moral beliefs, P-E misfit, and dropout). We used DWLS estimation with robust means and variance adjustment to conduct the CFA [48]. The model fit was satisfactory, χ^2^(60; N = 245) = 113.887, *p* < 0.001; CFI = 0.969; RMSEA = 0.030; and SRMR = 0.080 [49]. The standardized factor loadings of the observed variables (i.e., items) were all significant at *p* < 0.001, converged on the respective latent factors, and ranged from 0.223 to 0.959 (mean = 0.523). Correlations among latent variables were all statistically significant at *p* < 0.001 and in the expected directions, i.e., SDO and individualizing foundations = −0.510; SDO and misfit = 0.394; SDO and drop out = 0.123; individualizing foundations and misfit = −0.551; individualizing foundations and drop out = −0.251; and P-E misfit and drop out = 0.391. All correlations between latent factors were less than 1, and therefore achieved statistical discriminant validity [50].

### 3.2. Descriptives and Correlations

Descriptive statistics and bivariate correlations of the variables are reported in Table 1. As observed, SDO was significantly and negatively correlated with the endorsement of individualizing moral foundations (*r* = −0.416, *p* < 0.001) and significantly and positively correlated with both perceived P-E misfit (*r* = 0.286, *p* < 0.001) and drop out intention (*r* = 0.141, *p* < 0.05). The endorsement of individualizing moral foundations was significantly and negatively correlated with both perceived P-E misfit (*r* = −0.384, *p* < 0.001) and drop out intention (*r* = −0.217, *p* = 0.001). Finally, perceived P-E misfit was significantly and positively correlated with drop intention (*r* = 0.297, *p* < 0.001).

### 3.3. Serial Mediation Analysis

**Figure 2:**
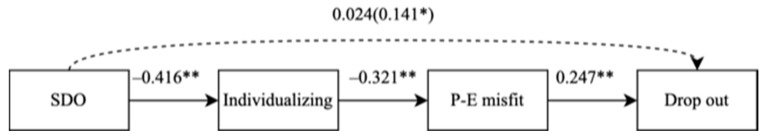
A serial mediation model showing the effects of SDO on drop out intention sequentially via individualizing moral foundations and P-E misfit. Note. All coefficients are standardized. Single-starred associations are significant at the *p* ≤ 0.05 level. Double-starred associations are significant at the *p* ≤ 0.001 level. The total effect is inside the parentheses. The dotted line indicates that an indirect effect of SDO on drop out is found.

We conducted a serial mediation analysis (PROCESS Macro; Model 6) [51] generating a 95% bootstrap percentile confidence interval of the indirect effects based on 5000 bootstrap samples. Results are presented in Table 2 and Figure 2. Standardized regression coefficients are also reported in Table 2. The endorsement of individualizing foundations was negatively and significantly predicted by SDO, *B* = −0.308, *SE* = 0.043, 95% CI [−0.392, −0.223], *p* < 0.001. Subsequently, endorsement of individualizing foundations negatively and significantly predicted perceived P-E misfit, *B* = −0.563, *SE* = 0.113, 95% CI [−0.786, −0.340], *p* < 0.001. Finally, perceived P-E misfit positively and significantly predicted drop out intention, *B* = 0.166, *SE* = 0.045, 95% CI [0.077, 0.255], *p* < 0.001. The total effect of SDO on drop out intention was significant *B* = 0.123, *SE* = 0.055, 95% CI [0.014, 0.232], *p* = 0.027. All indirect effects in the model are shown in Table 3. The completely standardized total indirect effect of SDO on drop out intention was significant, Effect = 0.117, BootSE = 0.048, 95% BootCI [0.023, 0.208]. Importantly, the completely standardized indirect effect of SDO on drop out intention, sequentially, via the endorsement of individualizing moral foundations and perceived P-E misfit, was also significant, Effect = 0.033, BootSE = 0.014, 95% BootCI [0.008, 0.061].

## 4. Discussion

Consistent with SDT [7,21], the present study highlights that SDO (i.e., personal support for group hierarchies) was associated with intentions to leave hierarchy-attenuating contexts that culturally support people in marginalized positions. This relationship was mediated by (i) a lack of endorsement of individualizing moral beliefs and (ii) a perceived attrition process toward the context (i.e., P-E misfit; [2,4]). Consistent with previous studies [14,35,36,37], our results confirmed that people higher on SDO hold few individualizing moral beliefs (Table 2). This finding suggests that people are motivated to reconcile their moral beliefs with their sociopolitical attitudes [34], which is consistent with the notion that people who score higher on SDO tend to view social relationships as competitive (i.e., zero-sum competition [13]). Consistent with this set of beliefs [23], low-status groups are viewed by those higher on SDO as “inferior” and therefore unworthy of moral consideration. Not surprisingly, SDO has been found to be associated with low empathy, toughness, and a desire for self-enhancement (vs. pro-social behavior) [12,14]. The absence of individualizing moral criteria contributes to the development of a structured mindset that reinforces a beliefs system oriented toward social dominance. [7,21,22]. Accordingly, we emphasize that SDO is confirmed as a meaningful sociopolitical orientation that acts as a self-guidance for behaviors aimed at producing asymmetrical intergroup relations [52].

In addition, our results highlight that individualizing moral beliefs did not mediate the relationship between people’s SDO and their intention to leave the hierarchy-attenuating context (Table 3, see pathway 1). This finding is noteworthy because it underscores the importance of the mediating role of the P-E misfit experience. Indeed, the perception of P-E misfit could be associated with the experience of feelings of “tension” caused by people’s assessment that their self-characteristics are not consistent with their actions in a hierarchy-attenuating environment [5,10]. Thus, our results suggest that it is primarily through a subjective assessment of P-E misfit that people are “primed” to think about viable solutions for managing their dissonance with a context that does not fit their orientations and belief systems (i.e., SDO and moral beliefs). In other words, only when people become aware of the P-E misfit do they seek ways to deal with it and achieve self-consistency (i.e., reduce cognitive dissonance) [53].

The results of the second mediation pathway (Table 3) support the arguments of SDT [6,28,30]: The attrition between sociopolitical attitudes and social norms shared in specific settings may lead people to consider dropping out. The act of dropping out could be seen as a strategy to cope with the perceived inconsistency [10]. People are willing to find viable solutions to reduce inconsistencies between beliefs, attitudes, and behaviors [53]. Interestingly, inconsistency has been found to be particularly frustrating for people with specific mindsets, i.e., closed, prejudicial, and eager for order and predictability [54]. The quickest way to resolve the dissonance caused by experiencing a P-E misfit may be to commit to maintaining a cohesive self-identity (i.e., one’s orientation, beliefs, and values) by rejecting prevailing social norms and considering leaving the context. Such attempts to achieve order and predictability in one’s life may also serve to protect one’s self-esteem, which has been found to be generally low in people higher on SDO [55].

Taken together (Table 3, pathway 3), the results of our serial mediation model confirmed our hypothesis, that SDO is associated with intention to withdraw from hierarchy-attenuating contexts because of the perceived inconsistency due to a P-E misfit. Our results extend previous findings [6,10,28] by suggesting that moral beliefs mediate this relationship. SDO can elicit lower individualizing moral standards that contrast with the moral bases normatively pursued in hierarchy-attenuating environments such as social work. Consistent with our hypothesis, people’s perceived P-E misfit and drop out intentions in contexts that value inclusive equality would directly predicted not only by their desire to support asymmetric intergroup relations (i.e., SDO), but also by whether SDO affects their moral standards.

## 5. Limitations and Future Perspectives

The present study has several limitations. The cross-sectional nature limits conclusions about the causal interpretation of the results. Although the direction of the associations between the study variables is theoretically and empirically supported by previous studies [8,10,35], further studies could confirm these patterns by implementing longitudinal methods. In the present study, we chose to analyze P-E misfit in a hierarchy-attenuating context (i.e., the social work environment), and the results should be extended with caution to other settings.

It is worth noting that most studies have focused on the psychology of high-SDO individuals, while evidence on the mindset of low-SDO individuals is limited [56]. A deeper understanding of the psychology of low-SDO individuals may also help to examine the P-E misfit dissonance experienced by such individuals in hierarchy-enhancing contexts. A promising line of inquiry might be to test whether low levels of SDO predict high individualizing moral concern, which in turn predicts P-E misfit and drop out.

Another topic to explore might be the other means used as strategies to reduce dissonance in hierarchy-attenuating and hierarchy-enhancing environments. People may cope with P-E misfit through means other than dropping out. For example, they can exploit a hierarchy-attenuating context by pursuing hierarchy-enhancing means (i.e., getting a higher salary, enhancing their self-image through success) [10]. Another way to address with the inconsistency of P-E misfit is by changing one’s own intergroup attitudes through adhering to hierarchy-reducing social norms (i.e., social pressure and conformity) [57].

Finally, the study sample was unbalanced in terms of gender, although this is a typical condition in social work contexts [9,26,58]. It has been reported that women tend to have lower levels of SDO [7], which is consistent with their choice to engage in a hierarchy-attenuating context [27]. Future studies can test the model in other types of samples not related to social work. These samples could be more balanced in terms of gender and age.

## 6. Practical Implications

The present study has important practical implications. First, the implications of possessing high SDO are consistent with the findings of other studies showing that individuals who possess traits associated with SDO, such as the dark triad and narcissism, are attracted to hierarchical settings and competitive selection procedures [59,60,61]. Competitive selection of human resources would also be consistent with the SDO-driven competitive worldview, with negative consequences for the groups and organizations involved. For example, competitive selection procedures could foster the incidence of selfish behavior, resulting in a consequent decrease in social welfare. Conversely, organizations could benefit from less competitive selection processes in order to increase social welfare [61] and reduce gender gaps [62]. Significantly, since P-E misfit fuels drop out intentions, organizations should aim to assess people’s socio-political P-E fit to promote their positive experience with the environment. The relevance is high in social work contexts, where professionals interact with people in highly vulnerable conditions and are exposed to the risk of professional burnout [63]. Positive individual-environment fit may also be influenced by the broader cultural context in which individuals are embedded, which may support the individual tendency to privilege hierarchies [64,65] and the morality underlying intergroup attitudes, with some cultures privileging respect for the rights and equality of individuals (i.e., individualizing focus) over others [15,19]. Therefore, an analysis of the broader cultural context is advisable to better understand the likelihood that individuals choose and remain in contexts that value social harmony, in response to social conformity processes [57].

## 7. Conclusive Remarks

In conclusion, the present study suggests that people’s support for intergroup hierarchies (i.e., SDO) is associated with a loss of moral concern for others (i.e., individualizing moral beliefs of care and fairness); this may lead to a perceived misalignment with hierarchy-attenuating social norms that are highly supported in contexts that value marginalized people (i.e., the poor, the disabled, immigrants, and the elderly). This sequential process increases the likelihood to drop out. These findings emphasize the value of psychological assessment of the social, political, and moral dimensions in the quest for positive functioning of organizations and communities.

## Figures and Tables

**Table 1:** Bivariate correlations and descriptive statistics.

	1	2	3	4	M (SD)
1. SDO	(0.84)				1.048 (0.778)
2. Individualizing	−0.416 **	(0.74)			4.991 (0.575)
3. P-E misfit	0.286 **	−0.384 **	(0.70)		1.275 (1.009)
4. Drop out	0.141 *	−0.217 **	0.297 **	(0.82)	1.419 (0.679)

Note: * *p* ≤ 0.05, ** *p* ≤ 0.001; In bracket (Cronbach’s α).

**Table 2:** Individualizing moral foundations, P-E misfit and drop out intention regressed on SDO.

Individualizing						
	*β*	*B*	SE	95% CI		*p*
				**LL**	**UL**	
SDO	−0.416	−0.308	0.043	−0.392	−0.223	<0.001
	R = 0.416, R^2^ = 0.173					
**P-E Misfit**						
	***β***	***B***	**SE**	**95% CI**		***p***
				**LL**	**UL**	
SDO	0.152	0.198	0.084	0.033	0.362	0.019
Individualizing	−0.321	−0.563	0.113	−0.786	−0.340	<0.001
	R = 0.408, R^2^ = 0.167					
**Drop out**						
	***β***	***B***	**SE**	**95% CI**		***p***
				**LL**	**UL**	
SDO	0.024	0.021	0.059	−0.096	0.138	0.723
Individualizing	−0.112	−0.132	0.083	−0.296	0.032	0.114
P−E misfit	0.247	0.166	0.045	0.077	0.255	<0.001
	R = 0.318, R^2^ = 0.101					

**Table 3:** Completely standardized indirect effects of SDO on drop out intention.

Pathway	Coefficient	BootSE	95% BootCI
(1) SDO → IND → Drop out	0.046	0.046	[−0.042; 0.142]
(2) SDO → P-E misfit → Drop out	0.038	0.025	[0.001; 0.096]
(3) SDO → IND → P-E misfit → Drop out	0.033	0.014	[0.008; 0.061]
Total	0.117	0.048	[0.023; 0.208]

Note: SDO = Social Dominance Orientation, IND = Individualizing moral foundations.

## Data Availability

Data are available upon request from the corresponding author.

## Appendix A

A number of studies have indicated that social work programs are among those that have as a core value the reduction of social hierarchies and inequalities among groups.

In particular, the policies and practices taught in social work programs promote the empowerment of socially, politically, and economically disadvantaged people (e.g., immigrants, the poor, the elderly, the disabled) in an attempt to reduce the disparities between these people and those with privileges. In light of what you have read above, please respond to the statements in the questionnaire by indicating how much you agree or disagree with each statement.
